# Supplementary material for: WordCommentsAnalyzer: A windows software tool for qualitative research
Source: F1000Res. 2018 Sep 4;7:536. Originally published 2018 May 3. [Version 2] doi: 10.12688/f1000research.14819.2 (PMC6137411; doi:10.12688/f1000research.14819.2)
Supplement: Supplementary file 3 [file f1000research-7-17614-s0002.tgz › b2a79496-4ada-47c4-9682-a99ce867836d.zip!/Eight Iranian Health Journals/Yafteh _41.docx]

© Yafteh  . This work is licensed under CC BY 4.0 (https://creativecommons.org/licenses/by/4.0/).

Volume & Issue: دوره 10 - شماره 4

Title: بررسی میزان تأثیر روش سم زدایی سریع با نالترکسون (NAD) بر ترک اعتیاد در معتادین مراجعه کننده به بیمارستان اعصاب و روان خرم آباد در نیمسال اول 1384

http://yafte.lums.ac.ir/article-1-97-fa.html

مقدمه: حدود 8% جمعیت بالغ کشور اعتیاد دارند و روزانه حدود 500 نفر به این جمعیت اضافه می شود. پیامدهای اقتصادی اجتماعی و بهداشتی بسیار سنگین چون طلاق، فحشاء، دزدی و قتل، ابتلای به ایدز و هپاتیت و ضرری معادل 29 درصد درآمد ملی از جمله عوارض اعتیاد برای کشورماست. امکانات محدود فعلی موجود در مراکز بستری و سرپایی درمان اعتیاد و روش های وقت گیر و هزینه بر موجود، باعث شده تا روش های ترک سریع اعتیاد از دیدگاه کارآیی، عوارض جانبی تحمل و عود اعتیاد هر روز بیشتر مورد توجه و امتحان قرار گیرند. مواد و روش‌ها: از فروردین تا شهریور ماه 1384، 140 مرد معتاد به مواد افیونی (هروئین و تریاک) که در دو گروه جداگانه همتاسازی شده بودند به ترتیب تحت سم زدایی سریع با نالترکسون و متادون قرار گرفتند. روند ترک اعتیاد در تمامی بیماران به مدت 3 ماه تحت نظر بود و در گروه اول کلونیدین، بنزودیازپین و نالترکسون همراه با درمان علامتی در 4 روز اول و ادامه ی درمان نگهدارنده با نالترکسون و در گروه دوم متادون تراپی به مدت یک ماه انجام شد . یافته‌ها: سن مددجویان بین 73 – 18 با میانگین 34 سال و بهره ی هوشی همگی حداقل نرمال بود. میزان موفقیت در روش سریع 55% و در روش متادون 50% بود. عود در روش سریع کندتر رخ داد و کمتر بود (45% در مقابل 50%). گرچه تفاوت از نظر آماری معنی دار نبود. میزان موفقیت بیشتر در روش سریع مربوط به نمونه های دارای سابقه ی اعتیاد کمتر و معتادین به هروئین بود که این مقادیر در روش متادون مربوط به نمونه های با سابقه ی اعتیاد بیشتر و معتاد به تریاک بود. در هر دو گروه ارتباط بین موفقیت در ترک با نوع اعتیاد، شغل، تأهل و سطح تحصیلات معنی دار نبود. مهمترین عارضه ی جانبی درمان افت فشارخون شریانی (در گروه سریع 10% و در گروه متادون 5%) بود. بحث و نتیجه‌گیری: روش سم زدایی سریع با نالترکسون با توجه به میزان موفقیت بالا، زمان کمتر و عوارض ناچیز می تواند روش مناسبی در سم زدایی افیون ها در زمان کمتر و حجم بیشتر داوطلبان ترک باشد.

Title: مقایسه تاثیر الانزاپین و سرترالین در بیماران مبتلا به اختلال شخصیت تحت درمان نگهدارنده با متادون

http://yafte.lums.ac.ir/article-1-98-fa.html

مقدمه: اختلال شخصیت مرزی یک اختلال ناتوان کننده است که 2 در صد جامعه را شامل میشود. درمانهای مختلفی برای آن پیشنهاد شده است. هر چه درمان بتواند طیف وسیعتری از علائم را پوشش دهد، مناسبتر است. در این اختلال ابتلاء به مواد مخدر نیز شایعتر است، که این امر موجب پیچیدگی علائم شده و بهبودی را دشوار تر می کند. مطالعه حاضر با هدف بررسی تاثیر الانزاپین و سرترالین در بیماران مبتلا به اختلال شخصیت تحت درمان نگهدارنده با متادون انجام گرفته است. . مواد وروشها: مطالعه حاضر یک کارآزمائی بالینی بوده است. 120 نفر زن و مرد پس از مصاحبه بالینی روانپزشک بر اساس معیارهای تشخیصی جهت اختلال شخصیت مرزی و تحت درمان نگهدارنده با متادون انتخاب و بطور کاملا تصادفی در دو گروه قرار گرفتند . یک گروه تحت درمان با الانزاپین با دوز 10-5 میلی گرم و گروه دیگر تحت درمان با سرترالین با دوز 100-50 میلیگرم قرار گرفتند. پرسشنامه 90% قبل از درمان ودر هفته های 4 ،8 و12 جهت کلیه واحدهای مورد پژوهش تکمیل شد. . یافته ها: مطابق کارآزمائی بالینی انجام شده در بیماران مبتلا به اختلال شخصیت مرزی تحت درمان نگهدارنده با متادون، سرترالین و الانزاپین در بهبود علائم افسردگی و اضطراب،کاهش حساسیت در روابط بین فردی و پرخاشگری و همچنین کاهش علائم وسواسی ،بدبینی و جسمانی کردن موثر بوده اند اگرچه در بسیاری از ایتم ها الانزاپین علائم را بیشتر تخفیف داده است. . بحث و نتیجه گیری: از آنجائی که نتایج مطالعه مبین تاثیر داروهای اولانزاپین و سرترالین در بهبودی علائم بیماران با اختلال شخصیت مرزی بود ، پیشنهاد می گردد از دارو درمانی توام با سایر درمانهای رواندرمانی جهت کنترل علائم اختلال استفاده شود .

Title: بررسی عوارض چشمی و عوامل مرتبط در بیماران تالاسمیک مراجعه کننده به بیمارستان ‏بوعلی سینای ساری در سال 87-1386‏

http://yafte.lums.ac.ir/article-1-99-fa.html

.‏ مقدمه: از آنجا که اطلاعات دقیقی در مورد عوارض بینایی در بیماران تالاسمی منطقه مازندران وجود ندارد دراین مطالعه ‏شیوع عوارض چشمی و عوارض مرتبط با آن در بیماران مبتلا به تالاسمی ماژور تحت پوشش بخش تالاسمی بیمارستان ‏بوعلی سینا ی ساری مورد بررسی قرار گرفت. ‏ .‏ مواد و روش ها: این مطالعه به روش توصیفی مقطعی وجامعه مورد مطالعه 60 نفر از بیماران تالاسمی ماژور تحت پوشش ‏بخش تالاسمی بیمارستان بوعلی سینای ساری بودند. بیماران از نظر وضعیت بیماری و عوارض آن از پرونده موجود در بخش ‏بررسی شده سپس بررسی حدت بینایی توسط اپتومتریست و با کمک چارت اسنل و بررسی ته چشم توسط پزشک انجام ‏شد. . ابزار مطالعه پتانسیل برانگیخته بینایی( ‏VEP‏ )دستگاه ‏TOENEES‏ ساخت ایتالیا بود که واجد قابلیت اخذ ‏VEP‏ و ‏P‏300 بود. یافته ها با نرم افزار ‏SPSS‏13 و استفاده از ابزار آمار توصیفی و تستهای آماری مناسب تحلیل گردید. ‏ .‏ یافته ها: مطالعه بر روی 60 بیمار که 33 نفر از آنها (55%) مذکر بودند انجام شد. در مطالعات چشمی 4 نفر (6/6%) از نظر ‏اپتومتری مشکل داشتند سه بیمار با اصلاح عیب انکساری دید نرمال پیدا نمودند. در معاینات اسلیت لامپ 3 بیمار (5%) ‏مبتلا به کاتاراکت به صورت ‏Inferior Posterior cortical cataract‏ بودند. 2 بیمار (3/3%) نیز رسوبهای دانه ای در ‏نواحی محیطی و سطحی عدسی ‏punctuate lens cortical opacity‏ داشتند. یک بیمار (7/1%) نیز ‏mild nuclear sclerosis‏ داشت. در معاینات با افتالکوسکوپ مستقیم و غیر مستقیم 4 بیمار (7/6%) تغییرات رنگدانه ای در ناحیه ماکولا و ‏یک بیمار (7/1%) تغییر در عصب بینایی به صورت ‏Left optic nerve head pit‏ داشتند . همچنین در ارزیابی ‏نوروفیزیولوژیک به صورت ‏VEP‏ هیچیک از بیماران یافته غیر طبیعی از نظر زمان تاخیر یا ‏latency ‏ و ولتاژ نشان ندادند.‏ .‏ بحث و نتیجه گیری: این پژوهش نشان می‌دهد که در بیماران ما مجموعاً 20% دچار عوارض چشمی بودند. عوارض یافت ‏شده در مطالعه ما می تواند ناشی از سمیت مستقیم دسفرال، توکسیسیتی آهن،و اثرات دارو در اتصال به مس و روی و خارج ‏کردن آنها از سلول‌های شبکیه و یا از عوارض خونسازی خارج مغز استخوانی تالاسمی باشد. مسلماً با استناد به یافته های ‏فوق معاینات و پیگیری مستمر این بیماران از لحاظ عوارض چشمی و تنظیم دوز دسفرال ضروری به نظر می رسد.‏

Title: سلول های استرومایی مغز استخوان و کاربرد آن در ضایعات عصبی

http://yafte.lums.ac.ir/article-1-100-fa.html

.‏ کلیات موضوع: در این مقاله مجموعه مطالعات آزمایشگاهی و بالینی صورت گرفته در زمینه استفاده از سلول های ‏استرومایی مغز استخوان جهت بهبود ضایعات عصبی، مورد بررسی قرار گرفته است .‏ تاریخچه: در مغز استخوان دو دسته سلول بنیادین وجود دارد: سلول های بنیادین خونسازو سلول های بنیادین غیر خون ‏ساز (استرومایی). بررسی های vitro In نشان داده است که سلول های استرومایی مغز استخوان قابلیت تمایز به سلول های ‏مختلف از جمله سلول های عصبی را دارا می باشد. از طرف دیگر مطالعات vivo In نیز نشان داده است که در پی تزریق ‏سلول های استرومایی مغز استخوان به بافت عصبی آسیب دیده، این سلولها ضمن یکپارچه شدن با بافت عصبی میزبان ‏وتمایز به سلول های عصبی، زمینه بهبود نسبی بافت آسیب دیده وبازگشت حرکات مختل شده را نیز فراهم می آورد. ‏همچنین بررسی ها نشان داده که علاوه بر تزریق مستقیم سلول های استرومایی در بافت عصبی آسیب دیده، از سایر روش ‏های غیر تهاجمی از جمله تزریق درون وریدی، تزریق درون بطنی و روش بزل کمری نیز جهت پیوند این سلول ها میتوان ‏استفاده نمود. ‏ .‏ مواد و روش‌ها: مطالعات صورت گرفته در خصوص تمایز سلول های استرومایی مغز استخوان به سلول های عصبی را می ‏توان در دو بخش مورد بررسی قرار داد: تمایز سلول های استرومایی مغز استخوان در In vitroو تمایز سلول های استرومایی ‏مغز استخوان در In vivo بررسی ها نشان می دهد که پیوند سلول های استرومایی مغز استخوان، روند ترمیم نقایص نورولوژیک را در مدل های حیوانی ‏سکته مغزی، ضربات مغزی، ضایعات نخاعی، بیماری پارکینسون و نیز ضایعات اعصاب محیطی بهبود می بخشد. ‏ .‏ بحث و نتیجه‌گیری: امروزه سلول های استرومایی مغز استخوان به جهت تهیه آسان از مغز استخوان بیمار و در نتیجه ‏عدم واکنش سیستم ایمنی به دنبال عمل پیوند، سرعت تکثیر زیاد سلول ها در محیط کشت، بیان ژن های مربوط به سلول ‏های عصبی، یکپارچه شدن با بافت عصبی میزبان پس از پیوند؛ به عنوان یکی از منابع سلولی مطلوب جهت بهبود ضایعات ‏عصبی مطرح می باشد. ‏

Title: بررسی میزان تحمل غیرطبیعی گلوکز و مقاومت به انسولین و بروز دیابت در بیماران ‏مبتلا به سندرم تخمدان پلی‌سیستیک در شهر شیراز

http://yafte.lums.ac.ir/article-1-101-fa.html

.‏ مقدمه: سندرم تخمدان پلی سیستیک شایع‌ترین اختلال هیپرآندروژنیک در سنین باروری می‌باشد. میزان شیوع آن ‏تقریباً 10-5 درصد خانمها در سنین باروری است. هدف این پژوهش بررسی میزان تحمل غیرطبیعی گلوکز، مقاومت به ‏انسولین عوامل خطرزای مؤثر بر بروز دیابت تیپ 2 در بیماران مبتلا به سندرم تخمدان پلی سیستیک و بررسی تحمل غیر ‏طبیعی گلوکز ومقاومت به انسولین می باشد.‏ ‏ ‏.‏ مواد و روش‌ها: پژوهش یک مطالعه توصیفی – تحلیلی است که به منظوربررسی تحمل غیر طبیعی گلوکز ومقاومت به ‏انسولین و بروز دیابت تیپ در بیماران مبتلا به سندرم تخمدان پلی سیستیک انجام شده است. نمونه‌های این مطالعه را 150 نفر ‏از بیماران واجدشرایط پژوهش تشکیل داده‌اند که با روش نمونه‌گیری آسان مبتنی بر هدف انتخاب شدند. از بین نمونه‌ها 125 نفر ‏آزمایشات را انجام دادند. ابزار گردآوری اطلاعات شامل پرسشنامه، ترازو، متر، آزمایش گلوکز ناشتا، انسولین ناشتا و تحمل ‏گلوکز با 75 گرم گلوکز خوراکی بوده است. جواب آزمایشهای قندخون براساس معیارهای 1999 سازمان بهداشت جهانی ‏سنجیده شده است. ‏ ‏ ‏.‏ یافته‌ها: 3/65%جامعه پژوهش در گروه سنی 29-20 سال قرار داشته است. اکثریت واحد ها (42%) دارای تحصیلات ‏ابتدایی و راهنمایی بوده،22% دارای سابقه دیابت و 3/71% تا کنون حاملگی را تجربه نکرده بودند. میانگین BMI‏ در افراد ‏مورد پژوهش 34/27با انحراف معیار 6/51 بود.نتایج نشان داد که 4/14 درصد افراد، اختلال گلوکز ناشتا (‏IGF‏) و در 4درصد، ‏دیابت شیرین دارند(‏mg/dl‏ 126≤‏FBS‏). نتایج قند خون دو ساعت بعددر 2/7موارد مختل بود(‏mg/dl‏ 199-140) همچنین ‏‏8/0%افراد دیابت شیرین (‏mg/dl‏ 126≤200).‏ .‏ بحث و نتیجه‌گیری: عوارض حاصل از هیپرآندروژنیسم و هیپراسولینیسم در زنان مبتلا به سندرم تخمدان پلی ‏سیستیک سلامت بیماران را تحت تأثیر قرار می‌دهد، آنها را در معرض بیماری‌های دیابت وقلبی و عروقی قرار می‌دهد. لذا ‏غلظت انسولین ناشتا و نسبت گلوکز ناشتا به انسولین ناشتا، مارکرهای مناسب برای تعیین مقاومت به انسولین در سندرم ‏سندرم تخمدان پلی سیستیک است. به همین دلیل انجمن دیابت آمریکا غربالگری اختلال تحمل گلوکز و دیابت تیپ 2 را در ‏مبتلایان به سندرم تخمدان پلی سیستیک توصیه کرده است.‏ ‏ ‏.‏

Title: میزان شیوع اختلال نقص توجه و بیش فعالی در کودکان دبستانی ‏ شهر خرم آباد‏

http://yafte.lums.ac.ir/article-1-102-fa.html

.‏ مقدمه: اختلال رفتاری کمبود توجه بیش فعالی ‏(ADHD)‏ یکی از شایعترین اختلالات روانپزشکی است که در کودکی ‏آغاز می شود و یک مسئله مهم بهداشت روانی جامعه به شمار می رود. آمار نشان می دهد 5-3% کودکان در سن مدرسه ‏مبتلا به ‏ADHD‏ می باشند. وجود مشکلات رفتاری از جمله ‏ADHD‏ اثرات مخربی بر روابط اجتماعی، تحصیلی، شخصیتی و ‏رفتاری افراد در کودکی و بزرگسالی دارد. بنابراین بر آن شدیم پژوهش حاضر را با هدف بررسی شیوع اختلال رفتاری کمبود ‏توجه – بیش فعالی در دانش آموزان دبستانی شهر خرم آباد انجام دهیم. ‏ ‏ ‏.‏ مواد و روش‌ها: این پژوهش یک مطالعه مقطعی است. جامعه آماری پژوهش شامل دانش‌آموزان مشغول به تحصیل در ‏سال تحصیلی 84-1383 در پایه‌های اول تا پنجم دبستانهای شهر خرم‌آباد می‌باشد. نمونه ها (945‏N=‏) از 16 مدرسه دولتی ‏و غیردولتی (8 مدرسه دخـترانه، 8 مدرسه پسرانه) بصورت‌ خوشه‌ای چندمرحله‌ای انتخاب شدند. ‏ ابزار گردآوری اطلاعات پرسشـنامه استاندارد شده ‏Child Symptom Inventories ‏4‏ (CSI‏4‏)‏ بود. که ابزاری برای ‏غربالگری شایع‌ترین اختلالهای روانپزشکی کودکان. پرسشنامه‌ها در دیداری جداگانه توسط والدین و معلمین تکمیل گردید. ‏سپس با تـوجه به نمره‌گذاری مواردیکه ‏ADHD‏ را نشان می‌دادند توسط روانپزشک معاینه بالینی شدند. سپس نتایج توسط ‏آمار توصیفی و آزمون ‏ مورد تجزیه و تحلیل قرار گرفت. ‏ ‏ ‏.‏ یافته‌ها: در 17/3 درصد افراد مورد پژوهش اختلال رفتاری ‏ADHD‏ گزارش شد که به ترتیب 40 درصد مربوط به اختلال ‏ADHD‏ از نوع بی‌توجهی، 3/33 درصد نوع بیش‌فعال و 6/26 درصد از نوع ترکـیـبی بود. ‏ADHD‏ در پسران بیشتر از ‏دختران گزارش دیده شد (9/4 درصد در برابر 5/1 درصد). بین جنس کودکان و اختلال ‏ ADHDارتـبـاط معـنـی‌دار وجود ‏داشت(005/0‏p<‏). دانش‌آموزان پایه‌های دوم و سوم دبستان بیشترین میـزان اختــلال ‏ADHD‏ و پایه پنجم کمترین میزان ‏را داشتند. ولی بین سن، میزان تحصیلات والدین، شغل والدین، میزان درآمد، پایه تحصیلی و مشکلات روانپزشکی در ‏خانواده ارتباط معنی‌دار وجود نداشت.‏ .‏ بحث و نتیجه‌گیری: با توجه به شیوع بالای اختلالات رفتاری از جمله بیش فعالی و کم توجهی در دانش آموزان ‏شناسایی این اختلالات در کودکان ضروری به نظر می‌رسد. ‏

Title: آنالیز تیتر آنتی بادی علیه هلیکوباکتر پیلوری درمقایسه با اندیکسهای آزمایشگاهی در کودکان 12-7 ساله مبتلا به آنمی فقر آهن

http://yafte.lums.ac.ir/article-1-104-fa.html

. مقدمه: کم خونی فقر آهن یکی از شایعترین کم خونی های رایج در دنیا بخصوص برای کودکان و بانوان در سنین بارداری می باشد. یکی از دلایلی که اخیرا به عنوان عامل مهمی در کاهش آهن خون به دلیل ممانعت از جذب گوارش آهن معرفی گردیده است هلیکوباکتر پیلوری می باشد. زیرا ایجاد گاستریت ناشی از این باکتری سبب ایجاد استعداد ابتلا به آنمی فقر آهن شده و این عمل بوسیله کاهش ترشح اسید هیدروکلریک و متعاقب آن کاهش جذب آهن صورت می گیرد. . مواد و روش ها: در100 کودک سنین 12-7 سال و با تشخیص قطعی آنمی فقر آهن ، میزان آهن سرم، فریتین ، TIBC و آنتی بادی IgG علیه هلیکوباکتر پیلوری بروش ELISA مورد بررسی قرار گرفت. در ضمن پرسشنامه ای شامل مشخصات دموگرافیکی و اطلاعات لازم از قبیل علائم بالینی به صورت محرمانه تکمیل گردید، سپس اطلاعات بدست آمده و نتایج آزمایشات مورد تجزیه و تحلیل قرار گرفت. . یافته ها: در بیماران مبتلا به آنمی فقر آهن فراوانی تیتر آنتی بادی IgG علیه هلیکوباکترپیلوری 90 درصد بوده است که از این میزان 80 درصد افراد دارای تیتر نشان دهنده آلودگی و 10 درصد تیتر با وضعیت تهاجمی بوده اند . آنالیز واریانس متغیرها نشان داده است که بین کاهش سطح آهن و فریتین سرم و افزایش TIBC، با افزایش IgG علیه هلیکوباکترپیلوری ارتباط معنی داری وجود داشت. . بحث و نتیجه گیری: در نهایت نتایج این مطالعه مشخص می کند که ابتلا به عفونت تهاجمی هلیکوباکتر پیلوری و کاهش جذب آهن می تواند در بروز آنمی فقر آهن در کودکان موثر باشد.

Title: بررسی ایجاد مقاومت علیه توکسو پلاسموزیس در مدل حیوانی با استفاده از آنتی ژنهای سطحی تاکی زوئیت توکسو پلاسما گوندیی

http://yafte.lums.ac.ir/article-1-105-fa.html

مقدمه: توکسوپلاسما گوندی یک انگل بیماریزای منتقله از راه غذا است که از طریق مصرف گوشت و عمدتا گوشت گوسفند و خوک آلوده- بوسیله اوسیست های دفع شده در مدفوع گربه، انسان را آلوده می سازد . آلودگی در انسان می تواند باعث ناهنجاریهای جنینی شدید،گرفتاریهای چشم ویا آنسفالیت گردد. ابتلا به توکسوپلاسموزیس در دوران حاملگی خصوصاً در دام اغلب منجر به سقط و لطمات اقتصادی قابل ملاحظه ای خواهد شد.هدف از این مطالعه بررسی این نکته است که آیا ایمونیزاسیون بوسیله آنتی ژنهای سطحی توکسوپلاسما گوندی می تواند باعث محافظت موش در برابر عفونت با این انگل شود؟ . مواد و روش‌ها: جهت انجام برنامه ایمن سازی از سویه بسیار بیماریزای RHدر مطالعه استفاده شد. آنتی ژنهای سطحی تاکی زوئیت ها که در محیط کشت تکثیر یافته بودند با استفاده از MEGA-10- یک دترجنت غیر یونیک - جدا شدند.گروههای 17تایی موشهای نر به دو صورت داخل صفاقی و زیر جلدی به فواصل ده روز،دو بار این آنتی ژن را دریافت کردند. 21 روز پس از دریافت آخرین دوز ، نمونه خون نیمی از موشها جهت انجام آزمایش IFAT گرفته شد و باقیمانده موشها تعداد 2000 تاکی زوئیت زنده و فعال سوش کشنده RH را به صورت داخل صفاقی دریافت کردند. . یافته‌ها: نتایج حاکی از افزایش تیتر آنتی بادی بعد از برنامه ایمونیزاسیون بود(05/0> p ) . نتایج بقای موشها پس از انجام آزمایشات (challenge) نیز نشان دهنده اختلاف معنی دار بین گروههای آزمایش (ID،IP) با گروه شاهد بود (05/0>p ). . بحث و نتیجه‌گیری: ادجوانت ها نقش مهمی را در برنامه های ایمن سازی ایفا می کنند.در هر صورت استفاده از ادجوانت های مناسب از قبیلISCOM مجموعه های محرک سیستم ایمنی(Immune Stimulating Complexes) و پروتئین های سطحی تاکی زوئیت توکسوپلاسما گوندی از قبیل P30 برای تحریک بهتر پاسخهای ایمنی سلولار و طبیعی پیشنهاد می گردد.

Title: اثر اسانس روغنی ساتوریا خوزستانیکا بر فعالیت و بیان ژن برخی از آنزیم‌های تنظیمی قند در کبد رتهای دیابتی و نرمال

http://yafte.lums.ac.ir/article-1-106-fa.html

. مقدمه: اثر اسانس روغنی ساتورجا خوزستانیکا جمزاد((SKEO نوع بومی بر فعالیت و بیان ژن برخی آنزیم های تنظیم کننده قند در کبد رتهای نرمال و دیابتی شامل گلوکوکینازGK))، گلیکوژن فسفریلازGP)) و فسفوانول پیرووات کربوکسی کینازPEPCK)) مورد ارزیابی قرار گرفت. . دهان به رتهای دیابتی و نرمال بمدت سه هفته خورانده شد. سطوح mRNA با استفاده از تکنیک Real time-PCRاندازه گیری شد. . یافته‌ها: غلظت قند خون رتهای دیابتی دریافت کننده SKEO در قیاس با رتهای کنترل دیابتی بطور معنی داری کاهش یافت(p<0. 001). فعالیت و سطوح mRNA آنزیم های GK و GP در رتهای دیابتی تحت تیمار با SKEO در قیاس با کنترل دیابتی بطور متوسط افزایش نشان داد گرچه این تغییرات معنی دار نیست. فعالیت PEPCK کبدی و سطوح mRNA آن در رتهای نرمال تحت تیمار با SKEO در قیاس با گروه کنترل نرمال بطور معنی داری کاهش یافت (05/0p<). سطوح افزایش یافته mRNA و فعالیت PEPCK رتهای دیابتی دریافت کننده SKEO در قیاس با گروه کنترل دیابتی بطور معنی داری کاهش می یابد (p< 0. 05). . بحث و نتیجه‌گیری: یافته ها اشاره دارند که گونه بومی SKEO به واسطه یک افزایش متوسط در مقادیر GK و GP بعلاوه یک کاهش قابل توجه در PEPCK در کبد رتهای دیابتی تحت تیمار ممکن است در عمل پائین آوردن گلوکز پلاسما سهیم باشد به نظر می رسد که این عمل در ارتباط با خواص آنتی اکسیدان SKEO باشد.

Title: بررسی اثر پیش شرطی سازی با هیپرکسی نورموباریک بر فعالیت آنزیم کاتالاز

http://yafte.lums.ac.ir/article-1-107-fa.html

چکیده مقدمه: پیش شرطی سازی به ایسکمی یکی از پدیده های درون زاد است که می تواند توسط عوامل مختلف و از مسیرهای مولکولی متفاوت در بافت های مختلف مانند مغز ایجاد شود. در این مطالعه اثر پیش شرطی سازی به واسطه هیپرکسی نورموباریک (HO) پیوسته و متناوب بر میزان، نقص نورولوژیک، حجم سکته مغزی و میزان فعالیت آنزیم کاتالاز مورد بررسی قرار گرفته است. . مواد و روش کار: رت ها در چهار گروه به صورت گروه های پیوسته (24 ساعت پیوسته) و متناوب (4 ساعت در روز به مدت 6 روز) در معرض HO و نورموکسی نورموباریک (RA یا هوای اتاق) قرار می گرفتند. هر گروه به سه زیرگروه تقسیم می شدند. زیرگروه اول، بعد از 24 ساعت، تحت جراحی انسداد شریان میانی مغز (MCAO) به مدت 60 دقیقه قرار می گرفتند و سپس 24 ساعت به آنها اجازه برقراری مجدد جریان خون داده می شد. زیرگروه دوم و سوم بنام زیر گروه شم (بدون MCAO) و گروه دست نخورده (بدون جراحی) برای بررسی اثر هیپرکسی نورموباریک بر میزان فعالیت آنزیم کاتالاز در نظر گرفته شده بود. . نتایج: یافته های ما نشان می دهد که HO متناوب و پیوسته در القای IT درگیر هستند. پیش درمان با HO پیوسته و متناوب نقص های نورولوژیک را بهبود می بخشد، حجم سکته مغزی را کاهش می دهد و میزان فعالیت آنزیم کاتالاز را به طور معنی دار افزایش می دهد. اثر هیپرکسی نورموباریک پیوسته نسبت به گروه هیپرکسی نورموباریک متناوب بر میزان فعالیت آنزیم کاتالاز بیشتر است. . نتیجه گیری: اگرچه برای شناخت مکانیسم حفاظت عصبی حاصل از هیپرکسی نورموباریک مطالعات زیادی لازم است، اما نتایج این تحقیق نشان می دهد که هیپرکسی نورموباریک آثار حفاظت عصبی خود را احتمالاً تاحدی از طریق افزایش میزان فعالیت آنزیم کاتالاز نشان می دهد.

Title: نقش استرس در افزایش فشار خون

http://yafte.lums.ac.ir/article-1-108-fa.html

. مقدمه: فشار خون بالا ناشی از استرس زمانی ایجاد می شود که فشار خون شخص در مطب یا مرکز درمانی بالاست ولی با استفاده از هولتر مانیتورینگ در حد نرمال است. این پدیده باعث می شود تا این افراد به درمانهای رایج ضد فشار خون پاسخ مثبت ندهند. . مواد و روش‌ها: این مطالعه مقطعی توصیفی با هدف بررسی میزان پرفشاری خون ناشی از استرس حضور بیمار در درمانگاه و مطب در بیمارستان شهدای عشایر خرم آباد در طی سالهای 85 تا 86 انجام شد. دویست بیمار مراجعه کننده به درمانگاه پس از اندازه گیری فشار خون توسط دستگاه جیوه ای استاندارد شده و با رعایت سایر شرایط در صورتی که میزان فشار خون بیمار در دوره های پرفشاری خون ملایم و متوسط بود به مدت 24 ساعت توسط دستگاه هولتر مانیتورینگ مدل در منزل نیز مورد بررسی قرار گرفتند. سپس میزان فشارها در دو روش با نرم افزارSPSS و با استفاده از آزمون آماری t مقایسه و مورد تجزیه و تحلیل قرار گرفتند. . یافته‌ها: در این مطالعه مقایسه فشار خون در درمانگاه و سپس اندازه گیری فشار خون 24 ساعته توسط هولتر نشان داد که 35% افرادی که در بدو ورود در درمانگاه در دو گروه پرفشاری ملایم و متوسط قرار گرفته بودند. فشار خون آنها در محدوده نرمال بوده و از این تعداد 70 نفر فشار خون بالا و 10 نفر مبتلا به دیابت و 49 نفر مبتلا به هیپرلپیدمی و 38 نفر سیگار می کشیدند. 65 نفر سابقه پرفشاری فامیلیال داشتند. آزمون آماری t اختلاف معنی داری بین میانگین میزان فشار خون سیستولیک و دیاستولیک در درمانگاه و مانیتورینگ 24 ساعته فشار خون نشان داد (001/p< ). . بحث و نتیجه‌گیری: در این مطلعه مقایسه میزان فشار خون در درمانگاه و همچنین میزان فشار خون در طی 24 ساعت مانیتورینگ نشان داد که 35% موارد که بطور روتین و با اندازه گیری فشار خون در درمانگاه و مطب تحت درمانهای دارویی و غیر دارویی قرار می گیرند عملا نیازی به درمان ندارند و فشار خون آنها در محدوده طبیعی می باشد. با توجه به اختلاف قابل توجه اندازه گیری فشار خون در مطب (درمانگاه)و فشار خون 24 ساعته در منزل استفاده از هولترمانیتورینگ در اکثر بیماران با فشار خون خفیف و متوسط ضروری است. . کلید واژه‌ها: فشار خون، قابل حمل، استرس، هولتر مانیتورینگ، W.C.H

Title: بررسی میزان اولئوروپئین در برگ درختان زیتون کشت شده در شهرستان خرم آباد

http://yafte.lums.ac.ir/article-1-109-fa.html

مقدمه: اولئوروپئین فراوانترین نوع از ترکیبات فنلی در برگ و میوه زیتون است و اثرات درمانی آن به عنوان یک آنتی اکسیدان قوی بخوبی شناخته شده است. میزان و نوع بیوفنلها در برگ زیتون بستگی به عواملی نظیر نوع رقم، آب و هوا، محل کشت، زمان برداشت و نیز روش استخراج دارد. در استان لرستان انواع گوناگونی از درختان زیتون کشت شده اند که اندازه گیری میزان اولئوروپئین در برگ این ارقام بعنوان منبعی غنی از این ماده می تواند بسیار با ارزش باشد. . مواد و روش‌ها: در این تحقیق برگ13 رقم مختلف زیتون پرورش داده شده در ایستگاههای تحقیقاتی شهرستان خرم آباد جمع آوری گردید. اولئوروپئین و ترکیبات وابسته پس از خشک و آسیاب شدن برگها طی دو مرحله 30 دقیقه ای در حمام فراصوت توسط حلال اتانل-آب (30: 70) استخراج گردید. برای اندازه گیری کمی اولئوروپئین در نمونه های استخراجی از دستگاه HPLC با ستون C-8 استفاده شد. . یافته‌ها: روش استخراجی مورد استفاده بطور موثر و کمی اولئوروپئین را از برگ زیتون استخراج نمود و بهینه سازی شرایط HPLC منجر به ارائه یک روش مطلوب برای جداسازی و اندازه گیری این ماده گردید. بررسی غلظت اولئوروپئین در 13 رقم زیتون مورد مطالعه نشان داد که اختلاف میزان این ماده در ارقام مختلف زیتون کاملا معنی دار می باشد. کمترین میزان اولئوروپئین در رقم کالامون به میزان 112.9 و بیشترین میزان آن در رقم سویلانا به میزان mg 7/235 در هر گرم گیاه خشک اندازه گیری شد. . بحث و نتیجه‌گیری: با توجه به ارزان و مقرون به صرفه بودن استخراج اولئوروپئین از برگ زیتون و بالاتر بودن میزان این ماده در ارقامی خاص ، با توجه به نتایج این تحقیق، می توان نسبت به تهیه اقلام دارویی حاوی در صد بالایی از اولئوروپئین بعنوان یک آنتی اکسیدان قوی و دارای خواص دارویی متعدد اقدام نمود.

Title: ارزیابی شیوع مارکرهای سرمی هپاتیت ب در کوهورت سنی متولدین 1368 شهرستان زابل

http://yafte.lums.ac.ir/article-1-110-fa.html

. مقدمه: ویروس هپاتیت B یکی از شایع ترین پاتوژن های مزمن در جهان به شمار می رود. حدود 350 میلیون نفر در سراسر جهان حامل مزمن هپاتیت ب هستند. بیش از سه چهارم عفونتهای هپاتیت ب در جهان در خاور میانه و افریقا واقع میشود. آمارمتفاوتی ازآلودگی به این ویروس ازایران گزارش شده است. با توجه به اینکه درآینده نزدیک طرح واکسیناسیون متولدین 1368 در شهرستان زابل (وکل کشور) انجام خواهد شد، و از وضعیت شیوع مارکرهای سرمی هپاتیت ب در این افراد و سنین کمتر اطلاعات و شواهد دقیقی در دسترس نیست. در این مطالعه وضعیت مارکرهای سرمی هپاتیت B در گروه سنی (متولدین 1368) در شهرستان زابل بررسی گردید. . مواد و روش ها: دراین مطالعه تعداد 458 نفر از جمعیت متولدین 1368 انتخاب شده وپس از تکمیل پرسشنامه از آنها نمونه خون گرفته شد. نمونه خون ازنظر مارکر های سرمی هپاتیت ب با استفاده از کیت Diapron ساخت کشور ایتالیا بررسی گردید ونتایج با استفاده از نرم افزار SPSS وارد کامپیوتر شده و بااستفاده ازتستهای آماری مناسب تجزیه وتحلیل شد. . یافته ها: نتایج مطالعه نشان داد 221 نفر (2/48%) مذکر و 237 نفر (8/51%) مونث بوده و 197 نفر (43%) ساکن شهر و 261 نفر (57%) ساکن روستا میباشند. از نظر قومیت 1/17 درصد آنها فارس، 67 درصد سیستانی و 9/15 درصد بلوچ بودند. در کل افراد مورد مطالعه 36 نفر (7/7%) HBsAb مثبت و 16 نفر (5/3%) HBsAg مثبت و 2 نفر (44/0%) HBcAb مثبت و 4 نفر (87/0%) HBeAg مثبت بودند. از کل افراد مورد مطالعه 73 نفر (9/15%) سابقه واکسیناسیون برعلیه هپاتیت ب داشتند که از بین آنها فقط 32 نفر (8/43%) HBsAb مثبت بودند. . بحث و نتیجه گیری: میزان 5/3% HBsAg مثبت برای گروه مورد مطالعه با توجه به اینکه هنوز در معرض ریسک فاکتورهای عفونت با HBV قرار نگرفتند زیاد بنظر میرسد و میزان 7/7% HBsAb مثبت نشان دهنده در معرض آلودگی بودن تعداد زیادی از جمعیت مورد مطالعه را بازگو مینماید. از طرفی فقط میزان 8/43% HBsAb مثبت بودن افراد واکسینه شده به دلیل عدم کارایی مناسب واکسن در منطقه نگران کننده است. بررسی موارد مذکور به مطالعات وسیعتری در منطقه نیاز دارد.

Title: سنتز و بررسی اثر بخشی آنالوگ های جدید داروی 4- کلرو آمودیاکین علیه دوفرم حساس و مقاوم به کلروکین پلاسمودیوم فالسیپاروم درمحیط In vitro

http://yafte.lums.ac.ir/article-1-111-fa.html

. مقدمه: مقاومت پلاسمودیوم فالسیپاروم به کلروکین به یکی از مشکلات عمده بهداشتی در کشورهای در حال توسعه تبدیل شده است. آمودیاکین یکی از ترکیبات 4-آمینوکینولین است که علیه بسیاری از فرمهای مقاوم به کلروکین موثر است ، اما بهر حال استفاده کلینیکی از آمودیاکین به جای کلروکین به شدت محدود شده است زیرا این دارو دارای اثرات هپاتوکسیکی و آگرانولوسیتوزی در مصرف کنندگان می باشد. . مواد و روش ها: در مطالعه حاضر آنالوگ های ایزومریک آمودیاکین در یک روش چهار مرحله ای تولید شده و علیه فرمهای پلاسمودیوم فالسیپاروم TM6 ( مقاوم به کلرکین ) و HB3 ( حساس به کلروکین ) بصورت in vitro آزمایش گردیده اند . انگل مالاریا بر اساس روش اصلاح شده Jensen و Trager برای کشت انگل پلاسمودیوم فالسیپاروم در گلبولهای قرمز انسان کشت داده شد.بر اساس این روش پلاسمودیوم فالسیپاروم در محیط کشت مداوم که حاوی گلبولهای قرمز گروه خونی O انسان در محیط کشت 1640 RPMIباضافه 10 درصد سرم AB انسانی و با استفاده از گاز ترکیبی که حاوی 3 درصد دی اکسید کربن،6 درصد اکسیژن و 91 درصد نیتروژن بود در 37 درجه سانتیگراد نگهداری شد. . یافته ها: چندین آنالوگ 4-کلرو آمودیاکین فعالیت ضد مالاریایی قوی به صورت in vitro علیه هر دو فرم مقاوم و حساس پلاسمودیوم فالسیپاروم نشان داده اند. آنالوگهای 5b ، 5c و 5i نه تنها در مقیاسهای نانومولار فعالیت ضد انگلی قوی داشته اند بلکه مقاومت متقاطع خیلی کمتری از خود نیز نشان داده اند. اثرات ضد پلاسمودیومم فالسیپاروم این آنالوگها علیه فرم حساس به کلروکین 3HB بسیار عالی تر از کلروکین بود و تا حدودی نسبت به آمودیاکین نیز ارجحیت داشته اند اگر چه با افزایش طول زنجیره جانبی این اثرات رو به کاهش می گذاشت مثلا در مورد آنالوگهای دی بوتیل و نیز آنالوگهای پیریدین ( 5g و (5j این اثرات قابل توجه بوده است. . بحث و نتیجه گیری: در یک فرایند چهار مرحله ای 10 آنالوگ مختلف کلروآمودیاکین ساخته شد که به صورت in vitro علیه گونه های مقاوم و حساس به کلروکین پلاسمودیوم فالسیپاروم اثرات امیدوارکننده ای نشان دادند. یکی از مشکلات 4-آمینوکینولینها داشتن واکنش مقاومتی متقاطع با کلروکین علیه فرمهای مقاوم به کلروکین پلاسمودیوم فاسیپاروم است که خوشبختانه بیشتر این آنالوگها چنین مقاومتی را در مقیاسهای بسیار ناچیزی نشان دادند. اگر چه چنین نتایجی دلگرم کننده است ولی مظالعات بیشتری لازم است تا اثرات ضد مالاریایی این آنالوگها علیه ایزوله های بیشتری از انگل مالاریا آزمایش گردد.
